# Morphology and arterial supply of the pyramidalis muscle in an Australian female population using computed tomography angiography

**DOI:** 10.1007/s00276-024-03471-1

**Published:** 2024-09-09

**Authors:** Craig L. Short, Tania N. Crotti, Kent Algate, Marc A. Gladman, Christen D. Barras

**Affiliations:** 1 School of Biomedicine, Faculty of Health & Medical Sciences, The University of Adelaide, Frome Road, Adelaide, South Australia, 5005 Australia; 2 Complex Benign Colorectal & Reconstructive Pelvic Surgery, King’s College, London, UK; 3 South Australian Health and Medical Research Institute, Adelaide, Australia; 4 Jones Radiology, Adelaide, South Australia Australia

**Keywords:** Pyramidalis muscle, Arterial supply, Vascular supply, Inferior epigastric artery, Prevalence, Morphology, Computed tomography angiography

## Abstract

**Introduction:**

The structure and function of the human anterolateral abdominal wall have been thoroughly described. However, there has been limited anatomical study of the pyramidalis muscle and its arterial supply. The aim of this study was to analyse the patterns of arterial supply to the pyramidalis in a female population.

**Methods:**

A retrospective study of 32 computed tomography angiography scans of the abdominal wall of adult women was performed to assess the prevalence (bilateral or unilateral presence, or absence), morphology (medial border height, base width and thickness) of pyramidalis and patterns of arterial supply.

**Results:**

Pyramidalis prevalence was bilateral in 75% of computed tomography angiography studies (24/32), unilateral in 6.3% (2/32) and absent in 18.8% (6/32). Of the five patterns of pyramidalis arterial supply observed and described in detail, the most frequent (68%, 34/50 of cases) originated from an exclusive muscular branch of the inferior epigastric artery. Origin from the pubic branch of the inferior epigastric artery was seen in 4% (2/50). There was a single case (2%, 1/50) of artery origin from a variant obturator artery, a common trunk with the pubic branch from the inferior epigastric artery, and from the muscular branch to rectus abdominis. The artery could not be defined in 22% (11/50).

**Conclusion:**

In this computed tomography angiography study of women, five patterns of Pyramidalis arterial supply were identified. In the majority of cases, the pyramidalis derived its arterial supply from an exclusive, isolated muscular branch of the inferior epigastric artery.

## Introduction

Pyramidalis is a small, triangular-shaped muscle of the anterior abdominal wall. Demarcated from the lower fibres of rectus abdominis by a thin fascial sheath [26], it ascends supero-medially from its place of origin on the pubic crest, the anterior pubic ligament and inguinal ligament, to its place of insertion on the linea alba (LA) [26]. To date, the precise function of pyramidalis remains unclear, however, some authors propose it acts as a tensor of the linea alba [7, 13, 15, 19], whilst others believe that it serves no purpose and may be regarded as a vestigial tissue [8]. A limited number of cadaveric studies from various ethnic populations and geographic origins have examined pyramidalis prevalence (bilateral presence, unilateral presence and bilateral absence [7, 11, 13, 15, 16, 23, 28] and morphometry [1, 7, 13, 15, 16, 23, 28]. However, no studies have investigated prevalence and morphology in living patients (in Australia) (Table 1).

**Table 1:** Comparison of morphometric data reported in the literature in a female population

Author	Number of Participants (*n*)	Number of Sides / muscles (*n*)	Age of Participants (Range, Years)	Specimen/Participant Preservation/Type	Medial Border Height – Left (mm)(SD) (95% CI)	Medial Border Height – Right (mm)(SD) (95% CI)	Base Width Left (mm)(SD) (95% CI)	Base Width Right (mm)(SD) (95% CI)	Thickness Right - Female (mm)(SD) (95% CI)	Thickness Left - Female (mm)(SD) (95% CI)
Das et al. [7]	8	12	35–60	Formalin-fixed cadavers	51.2 (13.8)	50.1 (13.6)	16.2 (4.2)	17.0 (5.0)	4.5 (1.3)	4.4 (1.3)
Hojaij et al. [13]	11	20	32–98	Fresh-frozen cadavers	63.8 (19.3)	64.2(17.8)	19.4 (3.0)	19.1 (3.5)	*NR	*NR
Kipkorir et al. [16]	11	18	*NR	*NR	64.7 (36.6)	62.2 (38.3)	*NR	*NR	*NR	*NR
Natsis et al. [23]	46	87	23–93	Fresh-frozen cadavers	65.6 (16.8)	61.8 (16.4)	15.5 (3.8)	15.0 (4.4)	*NR	*NR
Kaur et al. [15]	2	3	*NR	Formalin-fixed cadavers	47.8	48.7 (range, 47.8–49.6)	*NR	*NR	*NR	*NR
**Current Study**	26	50	34–65	CTA of living human participants	59.6 (16)	59.2 (17.5)	25.2 (5.1)	24.4 (6.1)	4.2 (1.7)	4.4 (1.6)

*NR – Not reported

Early-day anatomists have proposed various sources of arterial supply to the pyramidalis muscle including: as a branch of the cremasteric/external spermatic artery [2, 5, 23, 33], as a branch of the pubic division of the inferior epigastric artery [2, 5, 23, 33], and from a muscular branch of the inferior epigastric artery [6], whilst contemporary anatomical textbooks state the pyramidalis derives its arterial supply from branches of the inferior epigastric artery [10, 22]. However, in spite of these suppositions, there are no published image-based or anatomical studies investigating the arterial supply to pyramidalis [32].

Computed tomography angiography (CTA) is a diagnostic imaging method routinely used to investigate the adequacy, patency and patterns of arterial supply throughout the body. CTA has a reported sensitivity and specificity of close to 100% for identifying small calibre arteries of the anterolateral abdominal wall [3]. Indeed, CTA is the modality of choice for documentation of deep inferior epigastric artery (DIEP) patterns in female patients undergoing post-mastectomy breast reconstructive surgery [17, 21]. However, CTA has not been used to visualise the anatomical characteristics of pyramidalis, including prevalence, morphology and arterial supply. Therefore, the primary aim of this study was to investigate the arterial supply of the pyramidalis muscle in an Australian geographic origin. Secondary aims included determining prevalence (defined as bilateral presence, unilateral presence, and bilateral absence) and morphometric dimensions (including medial border height, base width, muscle thickness) to facilitate comparison with prior studies derived from other geographic origins.

## Methods

### Study population

A retrospective, analysis of CTA images was performed in a sample of 32 adult women of Australian geographic origin with mean age of 50.1 years ± 7.3 years (95% CI 47.5 years to 52.7 years). Participant height ranged from 1.57 m to 1.85 m, weight ranged from 63 kg to 103 kg, and body mass index (BMI) ranged from 22.3 to 41.8 Patients had undergone CTA between 2023 and 2024 at a private hospital in Adelaide, South Australia, This female cohort was chosen as the imaging protocol for their surgical workup provided an ideal retrospective opportunity for identification of the arterial supply to pyramidalis, prior to surgical body wall reconstruction surgery.

### CTA protocol

As a routine part of the imaging protocol, all patients were administered two pump sprays of vasodilatory sublingual glyceryl trinitrate (GTN) (Sanofi-Aventis, Australia, Pty. Ltd.), to improve vessel conspicuity [18] immediately prior to the scan. All CTA scans were performed on a Siemens Flash CT scanner (Siemens Medical Solutions, Erlangen, Germany) using dual source energy with kVp (kilovoltage peak) splits 100 kVp (tube A) and Sn-filtered 140 kVp (tube B). The pitch factor was 0.85, rotation speed 0.33 s, Qref mAs – 210 (Tube A @ 100 kVp), reconstruction kernel BV41 for small FOV reconstructions, and BV40 for standard FOV reconstructions. The slice thickness was 0.5 mm/0.4 mm increments for small FOV research reconstruction and 1 mm/0.8 mm increments for standard reconstruction as per manufacturer protocol. This study was granted ethics exemption by the Human Research Ethics Committee (reference number 35706).

### Image analysis

**Fig. 1:**
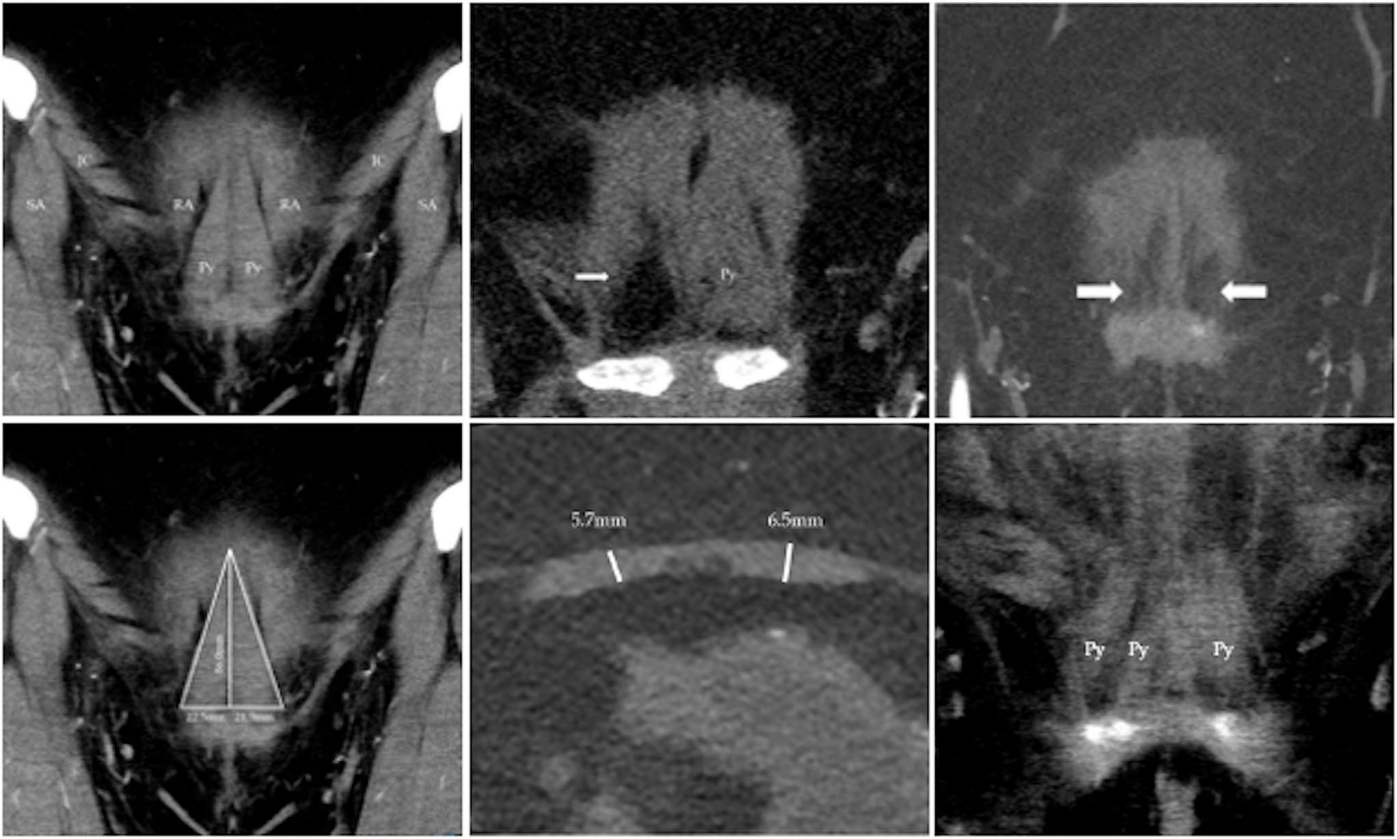
**a-f** CTA images. a: coronal view showing bilateral pyramidalis presence. Pyramidalis (Py), rectus abdominis (RA), sartorius (SA), inguinal canal (IC). b: coronal view showing unilateral pyramidalis presence. Pyramidalis (Py) and fatty tissue deposition in place of the right pyramidalis (white arrow). c: coronal view showing fatty tissue deposition in place of the pyramidalis, suggestive of pyramidalis absence (white arrows). d: coronal view showing measurement methodology for medial border height and base width. Pyramidalis (Py), rectus abdominis (RA), sartorius (SA), inguinal canal (IC). e: axial view showing measurement methodology for pyramidalis thickness. f: coronal view, showing pyramidalis duplicity (two pyramidalis muscles on the right and one on the left). Pyramidalis (Py)

The first (CS) and senior author (CB), a consultant radiologist of ten years’ experience analysed CTA images collaboratively for prevalence, morphology, and arterial supply of the pyramidalis muscle. Each scan dataset was analysed using 64-bit clinical imaging software Inteleviewer, version 4-18-1-P345 (Intelerad, Seattle, Washington, USA), and viewed using an EIZO radiology reporting monitor (RadiForce RX660, Japan). All measurements for this study were acquired using standard Inteleviewer tools. Three plane reconstructions of pyramidalis were created and tilted coronal plane views were used for muscle identification. Pyramidalis prevalence was established on bilateral (Fig. 1a) or unilateral (Fig. 1b) identification of an oblique lateral muscular border with obliquely orientated muscle fibres extending supero-medially from the pubic crest, distinguishable from the underlying vertically orientated fibres of rectus abdominis (RA) (Fig. 1a and b). Absence of pyramidalis was defined as an area of fat density at the location one would expect to visualise pyramidalis, with no discernible oblique muscle fibres (Fig. 1c).

### Morphometric analysis

Using coronal plane reconstructions, pyramidalis medial border height was measured from the point of origin at the pubic crest to the muscle apex, corresponding to its insertion on the linea alba (LA) (Fig. 1d). Pyramidalis base width was measured in the same coronal plane along the pubic crest origin from the most lateral aspect of the muscle to the pyramidalis medial border at the midline (Fig. 1d). Muscle thickness was measured in an axial plane, and tilted perpendicular to pyramidalis, to account for variation in muscle angulation between individuals. Pyramidalis thickness measurements were defined as the maximal muscle thickness obtained in an axial plane 5 mm superior to the pubic crest (Fig. 1e). This location was chosen to avoid measurement distortions from beam hardening artefact adjacent to bone [25].

### Arterial supply assessment

Arterial supply to pyramidalis was first assessed in the axial plane, identifying the characteristic inferiorly convex, recurving loop of the inferior epigastric artery and its subsequent branching pattern [14]. Next, each branch was followed to establish its course and termination using customised multiplanar reformats and maximal intensity projections to optimise arterial conspicuity. Findings were cross-referenced in all standard imaging planes. Each side of the patient was examined separately, with one complete analysis taking between 30 and 60 min. Line diagram reconstruction of the arterial pattern was independently performed by the anatomist for an inter-rater analysis.

### Statistical analysis

Statistical analysis was performed using SPSS statistical software version 28. The Shapiro-Wilk test for normality was conducted for continuous data. Mean and standard deviation (SD) values were reported. The paired samples T-Test was used to compare morphometric data for left pyramidalis compared to right pyramidalis muscles. The independent samples T-Test was used to compare the body mass index (BMI) of participants with bilaterally present pyramidalis muscles compared to bilaterally absent pyramidalis muscles. The Pearson’s Correlation test was used to assess for a correlation between participant height and pyramidalis medial border height.

## Results

### Prevalence

Bilateral prevalence of the pyramidalis muscle was 75% (24/32), unilateral prevalence was 6.2% (2/32) (1 left side, 1 right side) and bilateral absence was 18.8% (6/32) (Fig. 1a-c), resulting in a total of 26 patients with pyramidalis for further analysis. The mean BMI of participants with bilateral pyramidalis muscles was 27.2 ± 3.3 (95% CI 25.7 to 28.7) and for participants with bilateral absent pyramidalis muscles was 26.8 ± 2.6 (95% CI 24.0 to 29.6) No statistically significant difference in BMI was demonstrated between participants with bilaterally present pyramidalis muscles compared to bilaterally absent pyramidalis muscles (*p* = 0.793).

### Morphology

Measurements of pyramidalis medial border height and thickness were recorded in 50 pyramidalis muscles, (25 left pyramidalis muscles, 25 right pyramidalis muscles) in 26 patients (Fig. 1d, e). One participant displayed duplication of the right pyramidalis, with medial border distortion of both sides (Fig. 1f). Therefore, base width was recorded for 25 participants (48 pyramidalis muscles). Mean medial border height was 59.6 mm ± 16 mm (95% CI 53 mm to 66.2 mm) on the left and 59.2 mm ± 17.5 mm (95% CI 51.9 mm to 66.4 mm) on the right, Mean base width was 25.2 mm ± 5.1 mm (95% CI 23 mm to 27.3 mm) on the left and 24.4 mm ± 6.1 mm (95% CI 21.9 mm to 27 mm) on the right. Mean thickness was 4.4 mm ± 1.6 mm (95% CI 3.8 mm to 5.1 mm) on the left and 4.2 ± 1.7 mm (95% CI 3.5 mm to 4.7 mm). No statistically significant difference in medial border height (*p* = 0.808), base width (*p* = 0.355) or muscle thickness (*p* = 0.252) of the left pyramidalis muscle compared to the right pyramidalis muscle was demonstrated. The mean height of participants with symmetrical pyramidalis medial border height (*n* = 22) was 1.7 m ± 0.1 m (95% CI 1.6 to 1.7) No statistically significant correlation between participant height and pyramidalis medial border height was demonstrated (*p* = 0.496).

### Arterial supply

**Fig. 2:**
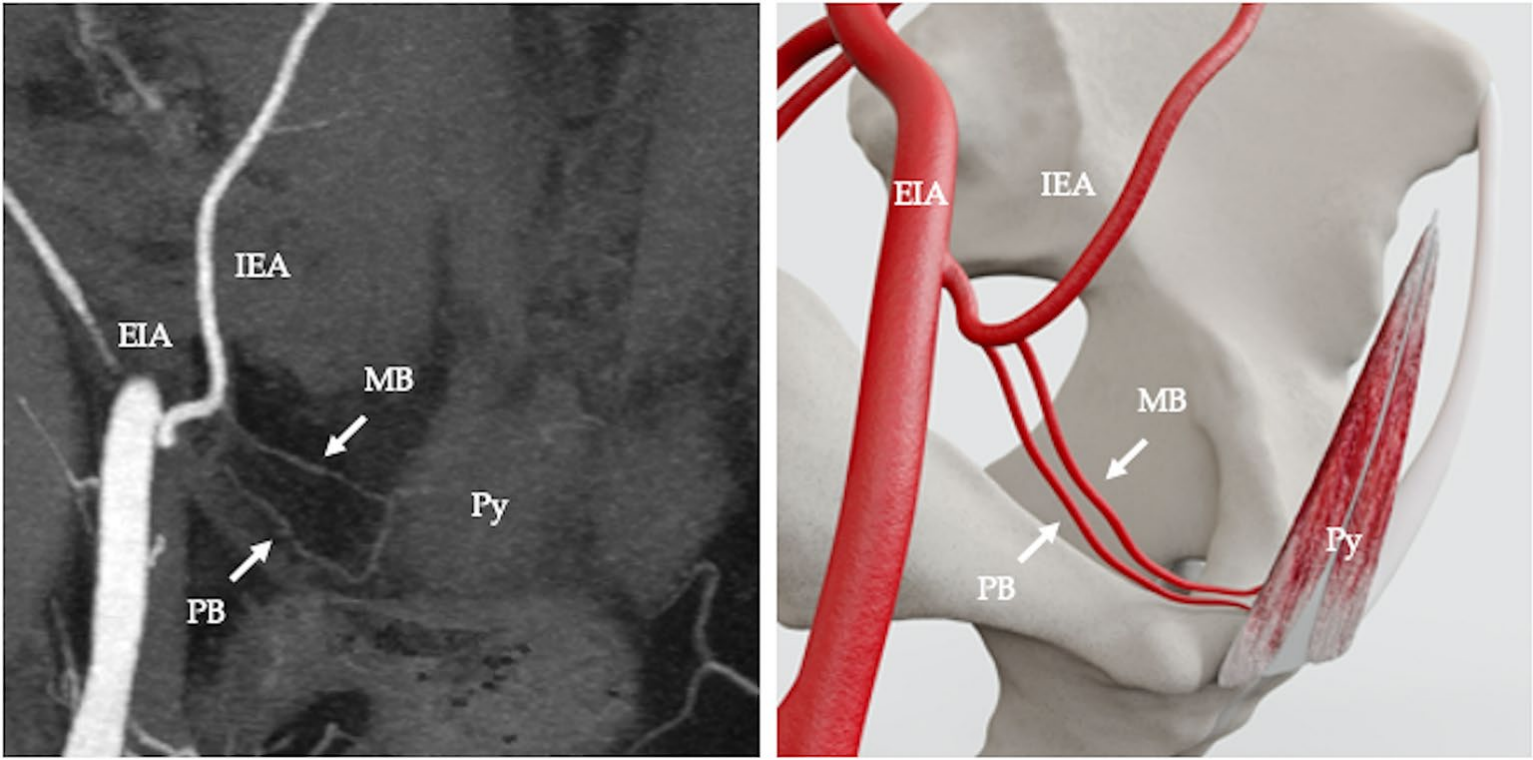
Computed tomography angiography (CTA), coronal view, showing arterial supply to pyramidalis from an exclusive branch from the IEA. External iliac artery (EIA), inferior epigastric artery (IEA), muscular branch of inferior epigastric artery (MB), pubic branch of inferior epigastric artery (PB) (**a**); diagramatic representation (**b**)

**Fig. 3:**
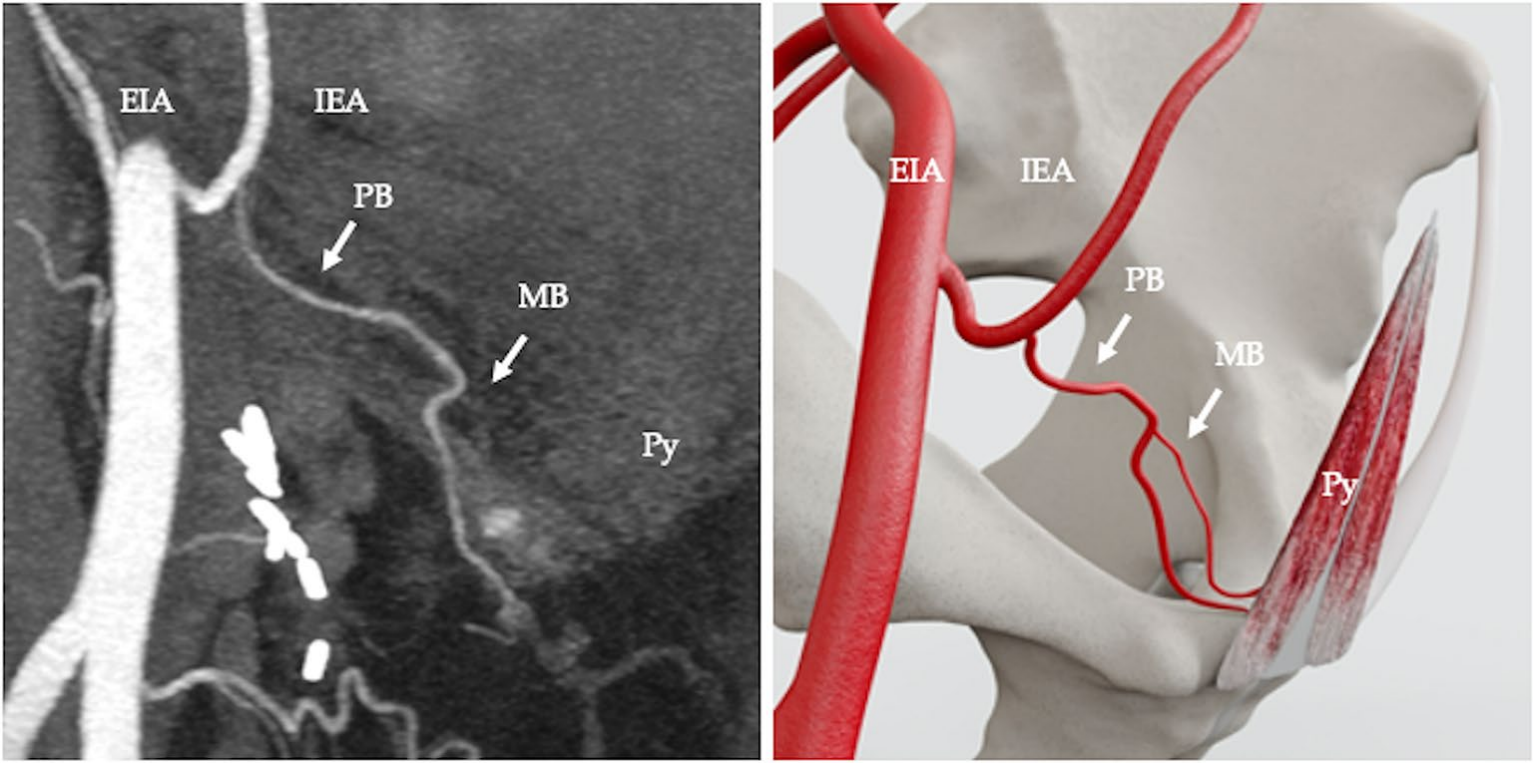
Computed tomography angiography (CTA), coronal view, showing arterial supply to pyramidalis from a muscular branch to pyramidalis (MB) originated as a branch from the pubic branch of the inferior epigastric artery (PB). Inferior epigastric artery (IEA), external iliac artery (EIA) (**a**); diagrammatic representation (**b**)

**Fig. 4:**
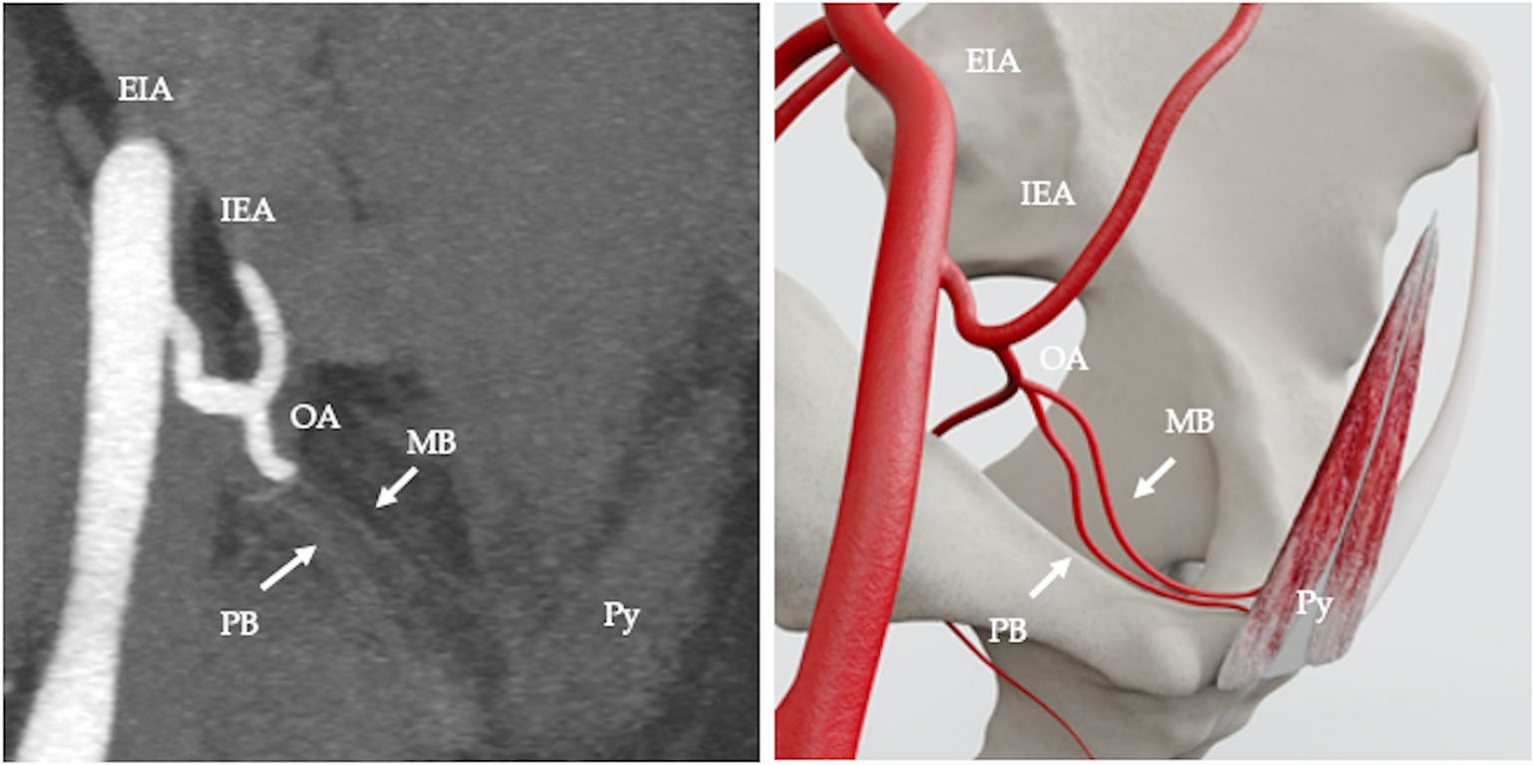
Computed tomography angiography (CTA), angled coronal view, showing arterial supply to pyramidalis from a muscular branch (MB) originating from a variant obturator artery (OA). External iliac artery (EIA), inferior epigastric artery (IEA), pubic branch originating from variant obturator artery (PB), pyramidalis (Py) (**a**); diagrammatic representation (**b**)

**Fig. 5:**
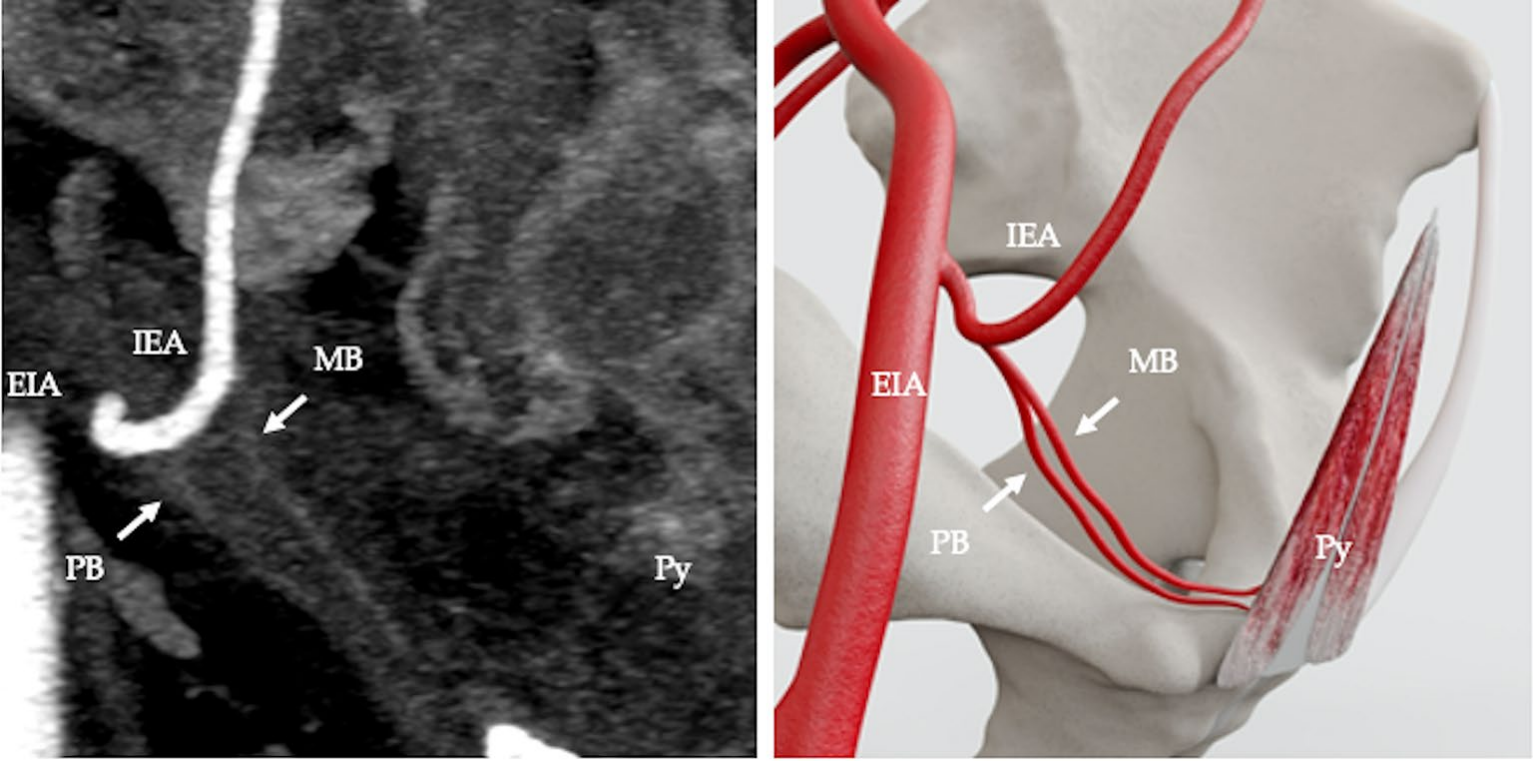
Computed tomography angiography (CTA), coronal view, showing arterial supply to pyramidalis from a muscular branch (MB) arising as part of a common trunk from the inferior epigastric artery (IEA) along with a pubic branch of the inferior epigastric artery (PB), external iliac artery (EIA) (**a**); diagrammatic representation (**b**)

**Fig. 6:**
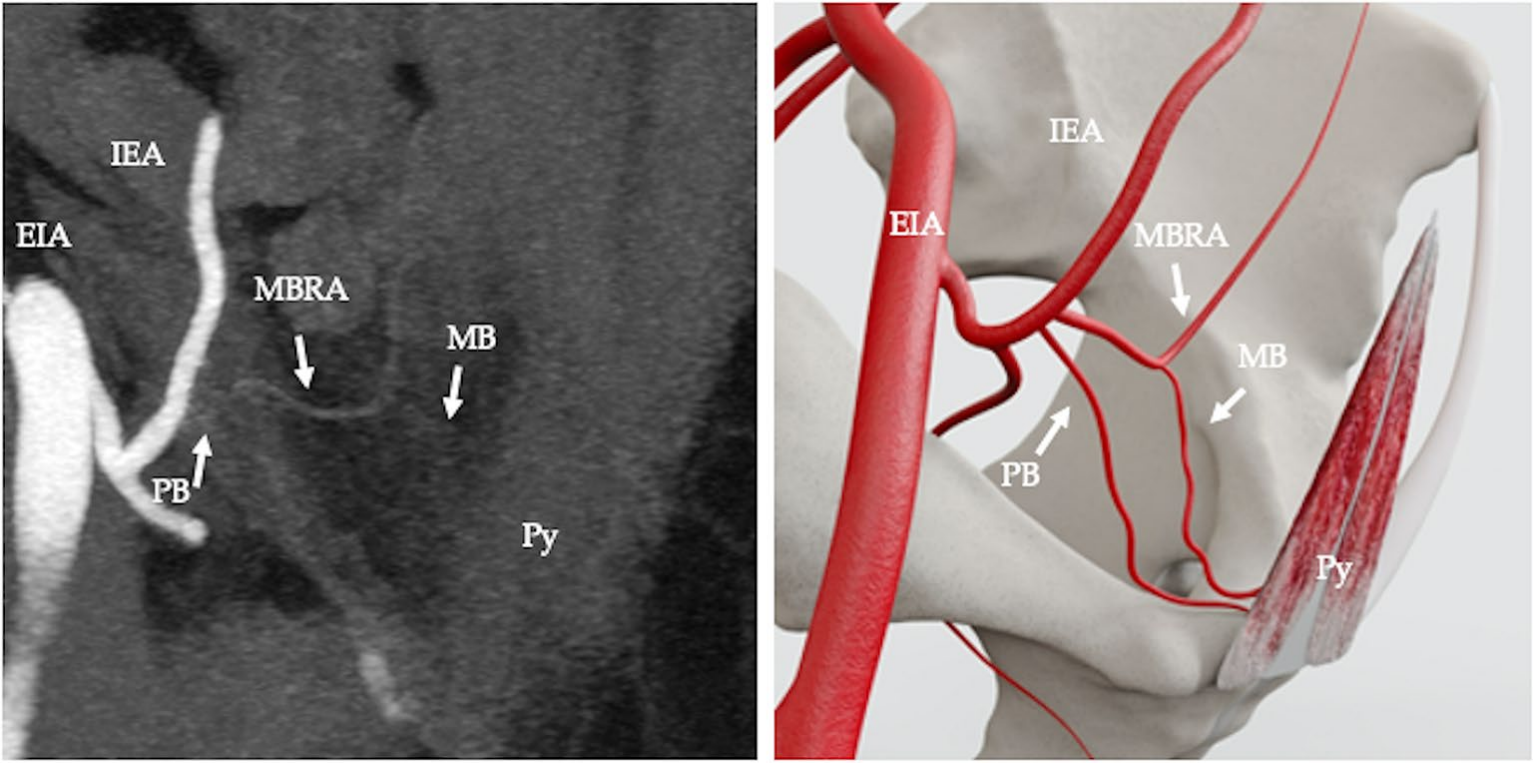
Computed tomography angiography (CTA), angled, coronal view, showing arterial supply to pyramidalis from a muscular branch to pyramidalis (MB) originating from a muscular branch to rectus abdominis (MBRA). Pubic branch (PB), inferior epigastric artery (IEA), external iliac artery (EIA) (**a**); diagrammatic representation (**b**)

The arterial supply pattern was examined in relation to 50 pyramidalis muscles. Pyramidalis derived its arterial supply from a muscular branch of the inferior epigastric artery (IEA) in 68% (34/50 cases) (Fig. 2). In 4% (2/50) of cases the muscular branch to pyramidalis originated from the pubic branch of the IEA (Fig. 3). In 2% (1/50) of cases the muscular branch originated from a variant obturator artery (Fig. 4) and in 2% (1/50) of cases the muscular branch originated as part of a common trunk, arising from the IEA (Fig. 5). In 2% (1/50) of cases the muscular branch to pyramidalis originated from a muscular branch to rectus abdominis (Fig. 6). In 22% (11/50) of cases the terminal branch to Py was either not seen or its origin and/or termination was not reliably discernible. Where discernible, there was 100% concordance with the arterial pattern recorded on radiologist-optimised CTA images and anatomist reconstructions.

## Discussion

This study identified the range of anatomical variation of the arterial supply to pyramidalis and reviewed its other morphologic characteristics using GTN-augmented CTA in live female participants for the first time. Additional novelty of this study includes the reporting of pyramidalis prevalence and morphological data in a female cohort of Australian geographic origin. Pyramidalis prevalence data varies across ethnic groups [7, 11, 13, 15, 16, 23, 28], supporting the proposed hypothesis of a genetic predisposition to presence or absence [23]. A systematic review of studies investigating the pyramidalis, hypothesised that the geographic origin in which the study was conducted to be highly relevant [4]. To date, the prevalence of pyramidalis has been investigated in studies from several geographic origins, including India [7, 15, 28], Greece [23], Brazil [13], Africa [16] and Japan [11], but the current study is the first to do so in a study population of Australian geographic origin.

A case of right-sided, unilateral pyramidalis duplication was identified in this study, a finding that has not been reported since 1848 [12]. Previous studies have reported that on occasion duplication can occur with two pyramidalis muscles occurring unilaterally or bilaterally totalling three and four pyramidalis muscles, respectively [15, 33]. Others have observed double pyramidalis muscles on both sides and although very small, all four muscles were distinct [12]. One study reports that the anatomist may rarely meet with three pyramidalis muscles in one subject [33], and others report that there are sometimes two pyramidales on one side, and one on the other [5]. Of 787 participants included in a systematic review and meta-analysis of the anatomical variations of the pyramidalis muscle [4], no cases of duplication of the pyramidalis were recorded.

Our findings for bilateral and unilateral prevalence of the pyramidalis muscle in Australian women of 75% (24/32), and 6.25% (2/32), respectively, is similar to the pooled prevalence estimates recently reported in a systematic review of a multiethnic population of 82.3% for bilateral prevalence, and 4.91% for unilateral prevalence [4]. Bilateral absence in the current study of 18.75% (6/32) is also similar to the pooled prevalence estimates for fresh frozen specimens reported as 11.9% in the said recent systematic review. The pooled prevalence estimates of 5.2% for bilateral absence in a population of human cadaveric specimens that had been formalin fixed, reported in the aforementioned systematic review and meta-analysis on the anatomical variations of the pyramidalis muscle differs from both their fresh frozen population and from our observations. This difference may result from the effect of formalin on cadaveric tissue planes, increasing the risk of tearing the fibres of pyramidalis as they adhere to the posterior aspect of the anterior rectus sheath. The pyramidalis fibres are frequently atrophic with some fatty replacement in the cadaver.

In this study, the most prevalent pattern of arterial supply to pyramidalis was derived from an exclusive, isolated muscular branch of the IEA. To our knowledge, the existence of such a vessel has been proposed by only one other anatomist [6]. In contrast to historically proposed origins of arterial supply to pyramidalis, this study found no examples of pyramidalis arterial origin from the cremasteric (external spermatic artery) branch of the IEA, or its female equivalent (the artery of the round ligament) [6, 20], or the pubic division of the IEA [2]. The present study population is exclusively female, with a mean age of 50 years, since the participant population consisted of female patients undergoing CTA ahead of breast reconstructive surgery using DIEP flap. The arterial supply to pyramidalis could not be reliably discerned by an experienced radiologist from CTA in 22% of cases, despite the 0.5 mm slice thickness and GTN-augmentation. Subjectively, this younger female cohort had no atheroma to interfere with the imaging interpretation, and it is likely the artery diameter was below the resolution of the imaging protocol in these cases. Knowledge of these arterial patterns from this study will aid evaluations of digital subtraction angiography and new technologies such as photon-counting CT angiography, since these imaging modalities may be more accurate than conventional CTA for very small artery identification [9, 30].

We found in our Australian female population, the prevalence of 75% (24/32), unilateral prevalence was 6.2% (2/32) (1 left side, 1 right side) and bilateral absence was 18.8% (6/32) and mean medial border height of 59.6 mm ± 16 mm (95% CI 53 mm to 66.2 mm) on the left and 59.2 mm ± 17.5 mm (95% CI 51.9 mm to 66.4 mm) on the right, to be consistent with recent publications [4]. Morphometric measurements have been reported in geographic regions including India [7, 15, 28], Greece [23], Brazil [13], Africa [16], and Pakistan [1] but none from Australia. Base width has been reported in the literature with great heterogeneity, ranging from 12 mm [15] and 19.9 mm ± 2.3 mm [4, 28], whilst the current study reported a mean base width of 25.2 mm ± 5.1 mm (95% CI 23 mm to 27.3 mm) on the left and 24.4 mm ± 6.1 mm (95% CI 21.9 mm to 27 mm) on the right. The variation may be due to the limitation of CT to precisely resolve the difference between connective tissue structures and adjoining muscle fibres leading to systematic overestimation of muscle base width. In addition, our study was based upon living, younger patients, rather than cadaveric studies, including older subjects. No statistically significant difference was found in medial border height (*p* = 0.808) or base width *p* = 0.355). These findings are consistent with prior published studies [23].

Only one other study has investigated pyramidalis thickness [7], however direct comparisons are compromised due to heterogeneity in methods between studies. The prior study [7], conducted analysis on 25 formalin fixed cadavers, including 17 males and 8 females of Indian geographic origin. They measured thickness at the mid-point of the muscle using measuring tape and digital vernier callipers, whilst the current study measured thickness using CTA images of 26 living female participants of Australian geographic origin. In the current study, the measurement was derived from an axial view, at a point 5 mm cephalad to the pubic crest using clinical radiology reporting software.

The pyramidalis is routinely used as a surgical landmark for accurate longitudinal incisions [7, 29]. Furthermore, the muscle is increasingly becoming a tissue of choice for many reconstructive procedures, including occlusion of fistulas [27], free flap procedures [32], colposuspension [31] and recurrent hernia repair [24]. The identification of pyramidalis and its arterial supply using CT in living patients may inform presurgical planning.

The authors acknowledge limitations within this study, specifically a small sample size of *n* = 32 (*n* = 50 pyramidalis muscles). In addition, our study population is exclusively female, with a mean age of 50 years ± 7.27 years, since the study is derived from a retrospective study of younger pre-surgical patient cohort. Whilst CT angiography of the lower limbs is also frequently augmented by vasodilators, which would allow inclusion of males, these studies are frequently compromised by atheroma, the indication for the majority of these tests, reducing the sensitivity for small vessel identification, and were not used in this study for this reason. Further studies with a larger sample size, mixed gender, age, and other ethno-geographic groups are recommended. Retrospective studies of digital subtraction angiography and new technologies such as photon-counting CT angiography are likely to improve vessel detection.

## Conclusion

Five principal variants of pyramidalis arterial supply were identified in Australian women on CTA. Pyramidalis prevalence and morphometric data in a population of Australian geographic origin aligns with the existing literature of other populations. An imaging example of pyramidalis duplication has been provided for the first-time, a potentially under-recognised variant in cadaveric studies. A more comprehensive understanding of the structure of pyramidalis will benefit from further studies in living patients using advanced imaging techniques. In the future, functional studies are needed to evaluate the purpose and importance of this diminutive muscle.

## Data Availability

Data used to support this study were derived from a private clinical database, which has restricted access. The deidentified images were accessed from this database following approval from the Human Research Ethics Committee- reference number 35706.
